# Implementing and applying the Ocular Trauma Score: the challenges

**Published:** 2015

**Authors:** Desirée C Murray

**Affiliations:** Lecturer in Ophthalmology: The University of the West Indies, St Augustine, Trinidad and Tobago, West Indies.

**Figure:**
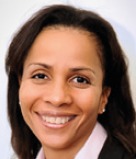
Desirée C Murray

Ocular trauma is a significant cause of unilateral blindness in the Caribbean in both adults and children.^1^,^2^,^3^ In Trinidad and Tobago, blunt ocular injury will typically account for around a third of all referrals from the Accident and Emergency department to the ophthalmology unit.^4^

The Ocular Trauma Score (OTS) aims to estimate a patient's visual acuity six months after an eye injury. A higher OTS score indicates a better visual prognosis.

The OTS was introduced at the Eric Williams Medical Sciences Complex, the main teaching hospital of the University of the West Indies, in 2012. The elements used to calculate the OTS (visual acuity, rupture, endophthalmitis, perforating injury, retinal detachment, relative afferent pupillary defect [RAPD]), were already routinely recorded during initial assessment of ocular trauma patients at the unit. It was expected that this would make the OTS easy to implement.

The OTS was first discussed during a postgraduate teaching session on ocular trauma. It was decided that the first on-call officer would calculate the score following initial assessment in the doctors' on-call examination room. A copy of the OTS was prominently displayed on the desk used for writing the notes, serving as a reminder to use it. It was decided that the score would be part of the presentation to the consultant on call and would be used to inform management decisions and discussion with the patients and their families.

Unfortunately, the use of the OTS was not sustained in the long term. Initially, there was inconsistent use of the OTS by the different ophthalmology trainees; the consultants also did not request the OTS score when the trainees presented each case to them. Then, when there was a change of staff at the junior and senior levels, its use was discontinued.

## Lessons learnt

Critical analysis of the OTS in an academic classroom environment (during the postgraduate teaching session), and displaying the OTS score prominently in examination rooms, helped to make clinicians aware of it and encouraged them to use it in their consultations with patients. However, this was not enough. The OTS should be implemented as unit policy and incorporated in all protocols and treatment guidelines in order to ensure its continued use. Capturing eye trauma patients' OTS scores for auditing purposes and analysing these data regularly will also help to demonstrate its usefulness.

It is worth the effort. The simplicity of the OTS allows medical and nursing staff with varying levels of experience to have a common understanding of prognosis. It is also an appropriate aid for counselling as it helps patients to understand their visual prognosis, which reduces unrealistic expectations. However, it is not a replacement for good clinical judgement – and the score is only applicable if all efforts are made to provide the correct management of the injury.

**Figure:**
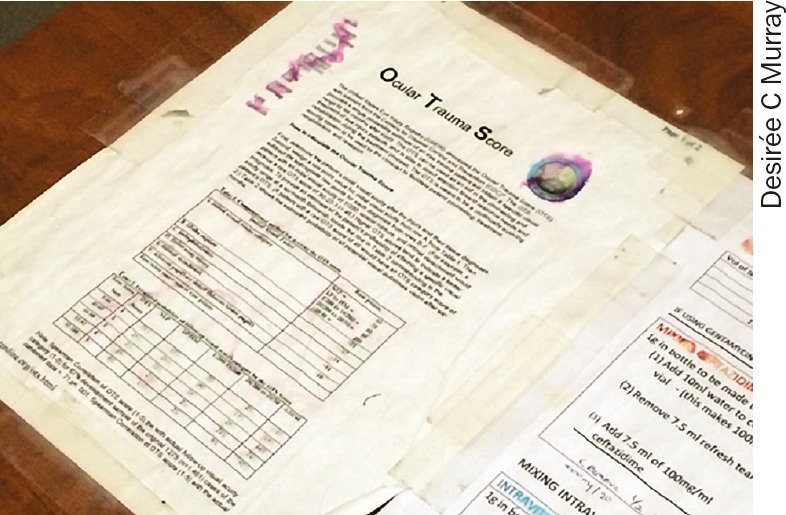
A copy of ocular trauma score was prominently displayed. WEST INDIES
